# Epilepsy Detection by Using Scalogram Based Convolutional Neural Network from EEG Signals

**DOI:** 10.3390/brainsci9050115

**Published:** 2019-05-17

**Authors:** Ömer Türk, Mehmet Siraç Özerdem

**Affiliations:** 1 Department of Computer programming, Mardin Artuklu University, Mardin 47500, Turkey; 2 Department of Electronics Engineering Dicle University, Diyarbakır 21100, Turkey; sozerdem@dicle.edu.tr

**Keywords:** Epilepsy, EEG, scalogram, Convolutional Neural Network, Continuous Wavelet Transform

## Abstract

The studies implemented with Electroencephalogram (EEG) signals are progressing very rapidly and brain computer interfaces (BCI) and disease determinations are carried out at certain success rates thanks to new methods developed in this field. The effective use of these signals, especially in disease detection, is very important in terms of both time and cost. Currently, in general, EEG studies are used in addition to conventional methods as well as deep learning networks that have recently achieved great success. The most important reason for this is that in conventional methods, increasing classification accuracy is based on too many human efforts as EEG is being processed, obtaining the features is the most important step. This stage is based on both the time-consuming and the investigation of many feature methods. Therefore, there is a need for methods that do not require human effort in this area and can learn the features themselves. Based on that, two-dimensional (2D) frequency-time scalograms were obtained in this study by applying Continuous Wavelet Transform to EEG records containing five different classes. Convolutional Neural Network structure was used to learn the properties of these scalogram images and the classification performance of the structure was compared with the studies in the literature. In order to compare the performance of the proposed method, the data set of the University of Bonn was used. The data set consists of five EEG records containing healthy and epilepsy disease which are labeled as A, B, C, D, and E. In the study, A-E and B-E data sets were classified as 99.50%, A-D and B-D data sets were classified as 100% in binary classifications, A-D-E data sets were 99.00% in triple classification, A-C-D-E data sets were 90.50%, B-C-D-E data sets were 91.50% in quaternary classification, and A-B-C-D-E data sets were in the fifth class classification with an accuracy of 93.60%.

## 1. Introduction

**Figure 1:**
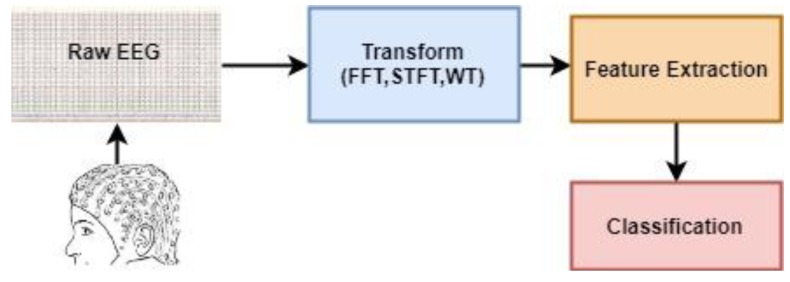
Common steps in conventional methods for the detection of epilepsy. EEG: electroencephalogram, FFT: fast fourier transform, STFT: short time fourier transform, WT: wavelet transform.

The brain, which is the center of all cognitive and sensory stimuli, also controls the vital functions in the body. This central unit has an excellent information processing function. In performing these functions, it produces complicated and complex biopotential signals [1]. These signals can be recorded using different methods. These records, called electroencephalogram (EEG), contain a lot of information related to the work of the brain and other organs of the body [1,2,3]. This information also allows us to obtain information about both cognitive and sensory stimulation [1]. EEG signals are used in this study because of their low cost and they contain lots of information. In addition, the abnormal activity of these signals is used for disease detection and contains important information for monitoring the disease. One of these diseases, which can be detected by EEG signals, is epilepsy [4]. It is estimated that this disease affects millions of people around the world [5]. Epilepsy is a disease that manifests itself in the form of seizures. Epilepsy seizure is an abnormal electrical activity that occurs temporarily in nerve cells [3]. From a clinical point of view, neurologists can examine the wave morphology of EEG signals in the detection of this disease and have an idea about the presence or level of the disease. However, the detection of this disease from EEG is based on the examination of long-term records, which is costly in time. Therefore, many studies have been proposed in the literature that may be helpful for specialized clinicians to detect automatic epilepsy from EEG. There are several important databases used in the proposed methods and they are publicly available. The database of the Department of Epileptology in the University of Bonn is at the top of these data sets [6]. Therefore, the Bonn data set was used in this study. There are many studies in the literature using the Bonn data set for epilepsy detection. These studies can generally be grouped under two classes. Which are:

(a) Studies Based on Conventional Methods
(b) Studies Based on Deep Learning Methods

(a) Studies Based on Conventional Methods: The EEG signals are by their nature unpredictable. When these signals are recorded, the information obtained first is time-amplitude information. However, EEG signals show potentials at different frequencies. For this reason, EEG signals are used to determine activity, while transformation methods that protect the structure of the signal and at the same time detect dominant (different) frequencies are prominent. In general, the main purpose of these methods is to move the EEG signal from the time-amplitude domain to the frequency-time domain. Examples of these transformation methods are Fast Fourier Transform (FFT), Short Time Fourier Transform (STFT), and Wavelet Transform (WT). Because of the length of the data to be used in the classification process after these transformations, feature methods are required to represent the characteristics and differences of the data set. In this scope, statistical parameters (mean, maximum, minimum Par), Hjorth parameters, spectral estimation methods can be shown among the frequently used methods for obtaining the content [7]. These features are then classified using a classifier. As the most widely used classifiers in this type of studies, K Nearest Neighbor (k-NN) Algorithm, Multilayer Artificial Neural Networks (MANN), Decision Trees Method, Support Vector Machine (SVM) can be shown [7]. In general, the path followed in the studies based on conventional methods is shown in Figure 1.

When we look at the literature study based on the Bonn database and conventional methods: Subasi (2007) used mixture of experts (ME) modular neural network architecture for controlled learning. In his study, he proposed a bi-cycle expectation-maximization (EM) algorithm to determine the epileptic seizure. Using discrete wavelet transform, the study decomposed the EEG signal into sub-bands. These sub-band frequencies are then input into the ME network as normal and epileptic. The study achieved a success of 94.5% [8]. 

Chandaka et al. (2009) have described a named pattern recognition technique that defines the SVM sequence with cross-correlation support. Certain techniques are used for the binary classification of EEG signals. A 95.96% classification success was obtained in their studies [9].

Li et al. (2013) suggested a new method based on empirical mode decomposition (EMD) and SVM. In their studies, first, the EEG signal was separated into the Intrinsic Mode Functions (IMFs) using the EMD method, and then the coefficient of variation and fluctuation index were obtained from these IMFs. These obtained features were evaluated in SVM classifier. In their study, they separated normal and epilepsy EEG separation by 98.00% sensitivity and 99.40% specificity [10].

Kaya, Y. et al. (2014), in their studies, obtained uniform and non-uniform features from epileptic EEG signals by using the 1D-LBP (One Dimensional Local Binary Pattern) method. In their classification with BayesNet, they found the accuracy of classification in the range of 93.00–99.50% and in the range of 92.80–99.50% with the non-uniform 1D-LBP in the features they obtained using the whole 1D-LBP method [11].

Xiang et al. (2015) suggested the method based on Fuzzy Entropy for the detection of epileptic seizures. The method first calculates the Fuzzy Entropy value of EEG signals from different epileptic states. They proposed the grid optimization method to train the obtained classification features using the SVM. They found normal and seizure EEG signaling accuracy of 100% in their study [12].

Kumar et al. (2015) divided the segments into the EEG signals before and during the seizure by applying a Gabor filter. The results obtained by applying the 1D-LBP method to the obtained segments have obtained a classification accuracy of 98.33% using the near neighbor algorithm [13].

Bhattacharyya et al. (2017), in their studies, analyzed the EEG signal by calculating multi-scale entropies. Quality scale (Q) based multi-scale entropy measurement is proposed to calculate the entropy of the EEG signal in different frequency bands. Q-based entropy (QEn) was calculated by separating the signal with the adjustable Q wavelength. In the study, k-NN entropy was calculated cumulatively from the sub-bands. The acquired features are assigned to the SVM. They found normal and seizure EEG signal and 100% classification accuracy [14].

Jia et al. (2017) used the complete ensemble empirical mode decomposition with adaptive noise (CEEMDAN) technique in their studies. They obtained various statistical features from their growth curve. These features are classified by random forest classifier. Ten-fold cross validation procedures were performed. They found the normal and seizure EEG signal to be 98.00% classification accuracy [15].

Zahra et al. (2017) used the multivariate empirical mode decomposition (MEMD) method to perform time-frequency (T-F) analysis in their studies. In the acquisition phase, Intrinsic Mode Functions (IMF) with lower frequency and noise were removed. Instantaneous frequency and amplitude information were obtained by applying Hilbert Transform to remaining IMFs. These obtained features were classified using artificial neural networks. They classified five different EEG datasets used in their studies with an accuracy of 87.20% [16].

Sharmila, A. et al. (2018), used discrete wavelet transform for the detection of epilepsy from the EEG signal in their studies. They obtained Shannon entropy and Approximate entropy (ApEn) values of sub-bands formed as a result of decomposition. They classified these values into SVM classifier. They found normal and seizure EEG signal to 100% classification accuracy [17].

Lu et al. (2018) used Kraskov Entropy based on the Hilbert Huang Transform (HHT) to obtain features. In the study, after decomposing the EEG signals into the internal mode functions, they calculated the Kraskov entropy applied on each internal mode function and the adjustable-Q (Tunable-Q) wavelet transform. They used the Least Squares Version of Support Vector Machine (LS-SVM) to classify these features. In their study, for different EEG classes, the classification success was found to be in the range of 81.96%–98.75% [18].

Ibrahim, S., Djemal, R., and Alsuwailem, A. (2018) proposed a different feature acquisition and classification technique to assist in the diagnosis of both epilepsy and autism spectrum disorder (ASD). First, the EEG signal is sub-banded using a discrete-wavelet transform (DWT). Standard deviation, band strength, Shannon entropy, and the largest Lyapunov base were obtained from these bands. Cross-correlation was also performed to measure the synchronization between the channels of the signal, which was not separated into the sub-bands. In their study, they found normal and seizure EEG signal using the different classifiers and the highest 100% classification accuracy [19].

**Figure 2:**
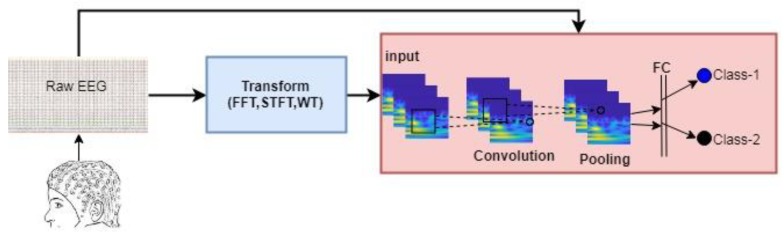
Steps in Deep Learning Methods. EEG: electroencephalogram, FFT: fast fourier transform, STFT: short time fourier transform, WT: wavelet transform, FC: fully connected layer.

(b) Studies Based on Deep Learning Methods: Currently, the methods in which few parts of the data are included in the classification are replaced by deep learning methods that quickly process very large data. Because the data in the conventional methods is represented by features, there is a great loss of data. However, thanks to deep learning networks, the EEG signal can be processed as raw. Deep learning mimics the human brain’s ability to observe, analyze, learn, and make decisions to solve particularly complex problems. Deep Learning has the ability to produce learning models and relationships beyond the adjacent affinities in the data. Therefore, the most important advantage of deep learning is the ability to perform the feature phase itself. In other words, deep learning networks process the data it receives and process the differences it finds on these feature maps. The path followed in the studies based on deep learning methods is given in Figure 2.

In the literature, the basic studies based on deep learning using the Bonn database are summarized below.

Ullah et al. (2018) divided the data set into four sub-segments of 1024. These sub-bands were then reduced to 50% overlapped and reduced to sub-bands using 512 window lengths. They classified the EEG data set thus obtained using the ensemble of Pyramidal One-Dimensional Convolutional Neural Network (P-1D-CNN) models. The seizure detection accuracy was 99.1% in their studies [20].

Hussein et al. (2018) first transformed EEG data were into a series of non-overlapping segments to reveal the correlation between consecutive data samples. Then, they used the Long Short Term Memory (LSTM) network and the Softmax classifier for classification to learn the high-level features of normal and seizure EEG models. Seizure detection accuracy was found in the range of 90.0–100% [21].

Yuan et al. (2018) transformed EEG records into EEG scalogram sequences using wavelet transform. Three different EEG features were obtained by using Global Principal Component Analysis (GPCA), Stacked Denoising Autoencoders (SDAE), and EEG segments, as global, channel-based and temporal features. Finally, by combining all of the features, the EEG was assigned to the SVM classifier for seizure detection. They found normal and seizure EEG signal, 100% classification accuracy [22].

Acharya et al. (2018) used EEG recordings (A, B, C, D, and E). In order to determine the normal, pre-seizure, and seizure classes, EEG recordings were applied to a 13-layer Convolutional Neural Network (CNN) algorithm. Each EEG data set consists of 100 × 4097 data points. 90% of the data set was used for training and 10% for testing. Thirty percent of the data used for the training was used as validation data during the training phase. The proposed technique provided 88.67% accuracy, 90.00% specificity, and 95.00% sensitivity success [23].

Considering the literature studies using conventional methods; it is known that many methods are used to obtain features from the data sets of these studies. In cases where a good performance cannot be obtained with the obtained features, it is tried to increase the performance by using different size reduction methods. This approach is very costly in terms of time and causes data loss.

In some of the deep learning-based approaches, direct training of feature vectors is provided. In this case, since the feature vector does not contain the characteristic of the data set, it can be seen that the desired success performance cannot be achieved. In some deep learning studies, raw EEG data were used directly for classification, but no good success performance was obtained. This deficiency seen in the literature motivated us to do this study. The stages of the method we proposed in this study are as follows: a) Frequency-time scalograms are obtained from raw EEG signals due to the success of deep learning networks in image processing area; b) Data sets are classified in CNN with different combinations. In this study, there are five different data sets (A, B, C, D, and E) and each data set is EEG records containing different activities. The images obtained from these records are evaluated on the CNN: Binary (e.g., A-B), triple (e.g., A-B-E), quad (e.g., A-C-D-E), and quintet (e.g., A-B-C-D-E) classification successes were obtained. All combinations of EEG classes with different activity were then classified. The aim of this approach is to show that each dataset with its own characteristic can be classified by the proposed method. On the other hand, unlike the studies in the literature, it is recommended to determine effective epilepsy detection method by evaluating the frequency-time scalograms images from the raw EEG signals without using any feature and size reduction method in the convolutional neural network that can learn their own. In addition, the success of the proposed method was evaluated by comparing the performance achievements with the other studies in the literature. Research findings have shown that the proposed approach is very effective in separating EEG signals.

## 2. Materials Methods

### 2.1. Dataset

The data set consists of five sets, A, B, C, D, and E. The characteristics of each cluster are given in Table 1.

**Figure 3:**
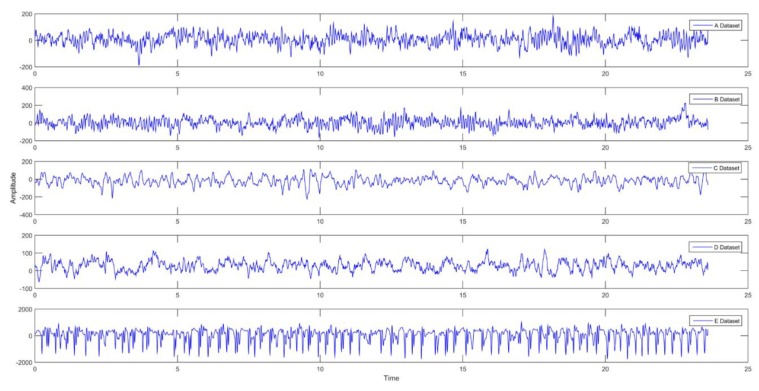
EEG data sets used in this Study.

EEG recordings were taken using the 10–20 international electrode positioning system. Each cluster consists of 100 parts with a single channel of 23.6 s. The EEG signals used were filtered through a 0.53–40 Hz bandpass filter. The sampling rate of the filtered EEG signals is 173.61 Hz. The sample signals for these five clusters are shown in Figure 3.

In this study, no pre-processing was applied to the data sets.

### 2.2. Methods

**Figure 4:**
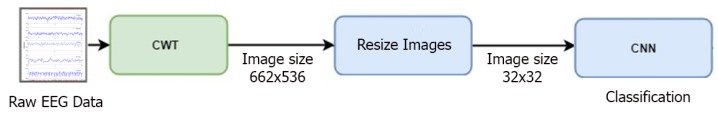
Followed in the study. EEG: electroencephalogram, CWT: continuous wavelet transform, CNN: convolutional neural network.

The EEG signals are inherently unpredictable. However, there are no obstacles in displaying these signals. For example, the instantaneous changes of EEG signals according to the unpredictable behavior in brain dynamics can be seen in different frequency bands. Therefore, in this study, Continuous Wavelet Transform (CWT) is used because it contains a lot of information in terms of frequency-time transformation and it can represent two-dimensional (2-D) EEG signal. The steps in the study are shown in Figure 4. The process steps are described below in detail, respectively.

#### 2.2.1. Continuous Wavelet Transform (CWT)

In the Wavelet Transform (WT), unlike the Short-time Fourier Transform (STFT), the function of the window undertakes a function called the main wavelet, which is both scaled and shifted during the conversion process. In this way, it provides long time interval windowing at low frequencies and short time interval windowing at high frequencies. In STFT, window sizes are constant and all the frequency information is analyzed at the same time-frequency resolution, while the CWT has the ability to split windows of different sizes, allowing it to best analyze the high and low frequency information in the time series [24,25]. WT It is a very effective method especially on non-stationary signals such as EEG. This method uses a small scale for high frequencies and a large scale for low frequencies to provide the best resolution [26]. The mathematical representation of CWT in continuous time is given in Equation (1):

(1) $$W_{x}\left(s,\tau \right)=\frac{1}{\sqrt{s}}\int_{-\infty }^{+\infty }x\left(t\right)\psi^{*}\left(\frac{t-\tau }{s}\right)\mathrm{dt}$$

where; W(s, τ) are the wavelet coefficients, x(t) is time signal, ψ(t) is the basic wavelet function conjugate, s is the scale and τ is the position parameter. In the study, the Morlet wavelet, which is more suitable than the other wavelet families, was used for the spectral analysis of non-stationary signals for continuous wavelet transformation [27,28].

The CWT contains a plurality of frequency values (components) for the analysis of continuous time signals, as it calculates by multiple expansions and the time offset of the wavelet. The local time frequency energy density measurement of this transformation is called a scalogram [29,30].

**Figure 5:**
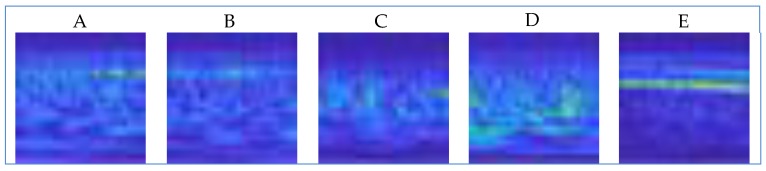
Sample scalogram changes of each segment for each set.

The CWT transformation was applied to the EEG data set using the Morlet Continuous Wave. Since a scalogram image was obtained from each segment, a total of 500 images (100 for A, 100 for B, 100 for C, 100 for D, and 100 for E) were included in the analysis. Examples of sample scalogram images for each cluster are shown in Figure 5.

#### 2.2.2. Resize Images

In this study, the frequency-time image was obtained by applying CWT to the raw EEG signal section of each class. The dimensions of these images are 662 × 536. These images were created using the cubic interpolation method to size 32 × 32 again to give the designed CNN input. These frequency-time images were classified into a CNN which is very popular today.

#### 2.2.3. Convolutional Neural Network (CNN)

ESA is an important deep learning approach with multiple layers trained in a solid way [31]. An ESA structure generally consists of three basic layers, namely convolution, pooling and a fully connected layer. These different types of layers play a role in different tasks. 

Convolution Layer: In the convolution process, the output value of a pixel is found as a weighted sum of the values of itself and of neighboring pixels. The weights matrix is called the convolution kernel or the filter. With the kernel filters used, the input image is convexed as a whole [32,33]. The basic equation of convolution is given below (Equation (2)):

(2) $$b_{k}=\sum_{n=0}^{N-1}x_{n}h_{k-n}$$

where; b, x, h, and N mean the output vector, the signal itself, the filter and the number of x elements, respectively. The applied h filter performs a windowing on the image and enables the identification of the features.

Generally, in an ESA network, activation is performed after convolution. In deep learning networks, the Rectified Linear Unit function (ReLU) is often used for activation [34]. The mathematical expression of ReLU activation function is given in Equation (3).

(3) $$f\left(x\right)=\{\begin{array}{c} 0\;\mathrm{if}\;x<0\\ x\;\mathrm{if}\;x\geq 0 \end{array}$$

Pooling Layer: In the pooling layer, it is aimed to reduce the feature map and reduce the number of parameters used in the network. The ESA pooling concept is a form of non-linear down sampling. In the pooling process, a set of non-overlapping rectangles is created from the input image and a maximum or average value is obtained from each sub-region (rectangle). With this method, it is possible to reduce the size of the property as required and to ensure the stability of translation [35]. Average pooling and maximum pooling are commonly used strategies. Maximum pooling was used in this study because of good results [36].

Fully Connected Layer: The features of the data pattern in this layer are converted into one-dimensional feature vectors [37]. Fully connected layers work just like conventional artificial neural networks. The most important disadvantage of the fully connected layer is that it contains too many parameters. Since they have too many parameters, the calculation load increases accordingly. 

#### 2.2.4. Structure and Training of the Proposed CNN

This study was carried out in the Pyhton environment by the Keras deep learning library. The obtained scalogram images were separated into 10 parts using the cross validation method. 20% of the data set used for training was used as validation data. In the CNN structure, 2 convolution layers and 2 pooling layers were used. The main parameters of these layers are given in Table 2.

**Figure 6:**
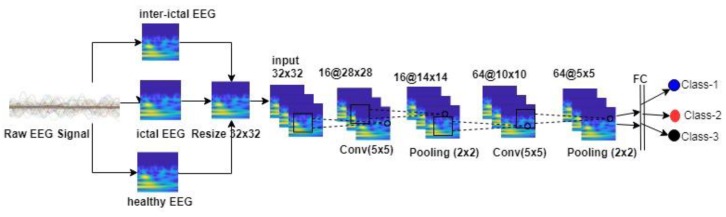
Recommended Two-Class process flow in CNN.

The steps of processing the EEG signal images in the network structure we have proposed in our study are given in Figure 6.

Images are given as 32 × 32 to ESA input. As a result of the operations in the first convolution layer, 16 feature maps are created and the images are converted to 16 @ 28 × 28. On the maxpooling layer, which is the next layer, the images are reduced to 16 @ 14 × 14. The image size from the second convolution layer is 64 @ 10 × 10 and 64 feature maps are obtained. This size is reduced to 64 @ 5 × 5 by maxpooling, which is the final layer. In the next layer, these images are subjected to vectorization and transferred to the fully connected layer. For the CNN, the learning rate is 0.001, the momentum is 0.9, the optimizer is Adadelta, the epoch number is 50 and the appropriate batch size is 4. 

All data were evaluated in system performance by using 10-fold cross validation for the reliability of the results obtained in the study. EEG scalogram images were divided into 10 equal parts in the CNN structure, 9 parts of these parts were used as training and the remaining 1 were used as test data. In order to avoid overfitting, 20% of the training data was allocated as validation data. The numbers of images used in these stages are given in Table 3.

#### 2.2.5. Performance Evaluation

All data were evaluated in system performance by using 10 cross-validation for the reliability of the results obtained in the study. Accuracy, sensitivity, specificity and f-score measurements were calculated for model performance evaluation. The values used for the calculation are given in Table 4.

Accuracy from model performance measurements; expresses the success of predicting the existing classes in the testing process of the model for medical diagnosis (Equation (4)). Sensitivity; expresses the correct estimating performance of the patterns in the test set (Equation (5)). Specificity; expresses the correct estimation performance of healthy patterns in the test set (Equation (6)). Finally, f-score refers to the measurement of the accuracy of the data being tested (Equation (9)) [38].

## 3. Results

In the study, frequency-time images obtained from EEG signals were evaluated in CNN structure. All combinations of EEG signals, each containing different activity, were compared. Classes compared in the study:

For binary data sets: A-B, A-C, A-D, A-E, B-C, B-D, B-E, C-D, C-E, and D-E

For triple data sets: A-B-C, A-B-D, A-B-E, A-C-D, A-C-E, B-C-D, B-C-E, B-D-E, D-C-E, and A-D-E

For quadruple data sets: A-C-D-E and B-C-D-E

For five data sets: A-B-C-D-E

In this section, the success performances of each class are given in tables.

Table 5 shows the performance measurements obtained in the double class classification of EEG records in the study.

In Table 5, different accuracy rates were found in the classification of data sets A and B with data sets D and E. However, A and B data sets showed the same characteristics with D-E data sets in binary classifications. In the study, it is seen that C and D data sets show the same characteristics in the classification with E data set. On the other hand, it is seen that C and D data sets can be separated at 80.00% accuracy. From this point of view, it was observed that the measurement region of the EEG signals before the seizure varied. In the case of reference, A or B datasets, the highest performance was obtained with the D data set, and in the E data set of the reference, the highest performance was obtained in the A and B data sets. Similarly, it was seen that C and D data sets showed the same characteristics in the classification with E data set. Based on this, it can be said that the brain region is not important in comparing the pre-seizure signal with the seizure moment signal. On the other hand, it can be said that the brain region is important in comparing pre-seizure signals (C or D) with healthy signal (A or B). In addition, in the separation of the healthy A and B datasets, it can be seen that the proposed method can significantly catch up with the difference in the closed eye state (95.50%). On the other hand; In comparison with the epileptic signal, the signal processed in the healthy and open eye was seen to be more successful than the closed eye signal (Example A-E, B-E). In addition, it was observed that the healthy and closed eye signal was compared to the pre-seizure epileptic signal and it was more successful than the healthy and open eye signal (Example A-C, B-C).

In the study, triple class classification performance is given in Table 6.

In triple classification performance: A-B-C 95.00%, A-B-D 96.67%, A-B-E 95.67%, A-C-E 97.00%, A-C-D 88.00%, A-D-E 99.00%, B-C-D 91.33%, B-C-E 98.67%, B-D-E 98.00%, and D-C-E 89.00%, were separated by the average accuracy (overall). In the classification of health signals with pre-seizure signals, it is observed that the closed eye signal (B data set) is better than the open eye signal. Another important issue in the study is that the performance of the signal with the seizure signal (E data set) of the healthy signals is lower than the signals before the seizure. The lowest classification performance in the triple classification was obtained from A-C-D data sets with 88.00%, while the highest achievement performance was obtained from A-D-E data set with 99.00%.

Table 7 shows the quaternary class classification performance.

In quaternary class performance, A-C-D-E and B-C-D-E were classified with an average of 90.50% and 91.50% accuracy (overall), respectively. In this classification, it is seen that A and B data sets show almost the same characteristics in classification with other classes. In the same way, the data sets A, B, and E in (A-C-D-E) and (B-C-D-E) were separated at the same sensitivity ratios.

The classification performance in which all classes are included is given in Table 8.

In the five-class classification, the A-B-C-D-E was found to have an average of 93.60% accuracy. In Table 8, 99.36% accuracy was obtained from the highest E data set.

## 4. Discussion

Very successful results are obtained with deep learning architecture which can be applied to many areas today. Therefore, deep learning has become a central position in machine learning and pattern recognition. In the world of science, it is predicted that deep learning networks, which are adding new methods every day, will achieve the desired success in many areas in the future. In addition, robust inference through deep learning is predicted to improve the reliability of clinical decision support systems. By using deep learning networks, more successful results can be obtained in separating multiple classes.

In this study, a method that can be used as a clinical decision tool in the detection of Epilepsy in EEG signals is proposed. The performances obtained by the proposed method compared to other methods used in the literature are given in Table 9.

As can be seen in Table 9, it was concluded that the proposed method could provide an important classification accuracy compared to other studies. Currently, deep learning networks have achieved great success in image processing. Therefore, in our study, frequency-time images of five different EEG signals with different activities using CWT were evaluated in CNN. The results are compared with many studies in the literature. 

In the literature studies, features were obtained by using different methods (Table 9). In some studies, it is aimed to increase the success by using size reduction methods. In addition, in most studies only certain classes could be compared. There are two reasons for this. The first is the detection of seizures and pre-seizures (pre-ictal) from EEG signals. The second is that the characteristics of each EEG data are different. Because, in order to achieve a good success performance in different classes, different features must be used. This is not a practical solution for the classification stages of EEG signals. In our study, unlike the studies conducted;

- No feature has been obtained from the EEG dataset. At the same time, no size reduction method was used.
- Frequency-time scalograms of raw EEG data were evaluated directly in the CNN structure.
- The comparison of all classes was performed to evaluate the success of the proposed method. It has been found that the proposed method can successfully distinguish each data set with its own characteristic.
- It has been observed that the method used in the study provides a much better success than the methods used in the literature, especially when the data set diversity increases.

It has been observed that the method we offer has very good results in distinguishing EEG signals. Therefore, if EEG signals are to be evaluated in CNN, we recommend to convert them to images by using various conversion methods, but also to not use methods such as gaining or decreasing the signal.

## Figures and Tables

**Table 1:** Clusters and Properties of Electroencephalogram Datasets Used in the Study.

A	B	C	D	E
Healthy	Healthy	Epilepsy Patient	Epilepsy Patient	Epilepsy Patient
Total of 100 segments	Total of 100 segments	Total of 100 segments	Total of 100 segments	Total of 100 segments
Duration of each segment 23.6s	Duration of each segment 23.6s	Duration of each segment 23.6s	Duration of each segment 23.6s	Duration of each segment 23.6s
Eyes open recording	Eyes closed recording	Pre-Seizure, recording from the hippocampal half sphere	Pre-seizure, record from the epileptic area	Record during the seizure

**Table 2:** Main Parameters of Convolutional Neural Network Model.

Layer	Filter Size	Number of Filters	Number of Neurons	Stride
Conv-1	5 × 5	16	-	1
MaxPooling	-	-	-	2
Conv-2	5 × 5	64	-	1
MaxPooling	-	-	-	2
FullyConnected	-	-	1000	-

**Table 3:** EEG Scalogram Image Numbers Used for the CNN in Each Fold Cross-Validation.

Data Set Taken into Consideration	Total Number of Images	Number of Images Used for Training	Number of Images Used for Validation	Number of Images Used for Testing	Number of Classes at CNN Output
Two	200	144	36	20	2
Three	300	216	54	30	3
Four	400	288	72	40	4
Five	500	360	90	50	5

**Table 4:** Confusion Matrix and Model Performance Criteria.

Predicted Class			
Original		Class = 1	Class = 0
	Class = 1	True Positive (TP)	(False Positive) FP
	Class = 0	(False Negative) FN	(True Negative) TN
(4) $$\mathrm{Accuracy}=\frac{\mathrm{TP}+\mathrm{TN}}{\mathrm{TP}+\mathrm{FP}+\mathrm{FN}+\mathrm{TN}}$$			
(5) $$\mathrm{Sensitivity}=\frac{\mathrm{TP}}{\mathrm{TP}+\mathrm{FP}}$$			
(6) $$\mathrm{Specificity}=\frac{\mathrm{TN}}{\mathrm{FP}+\mathrm{TN}}$$			
(7) $$\mathrm{Precision}=\frac{\mathrm{TP}}{\mathrm{TP}+\mathrm{FP}}$$			
(8) $$\mathrm{Recall}=\frac{\mathrm{TP}}{\mathrm{TP}+\mathrm{FN}}$$			
(9) $$F-\mathrm{Score}=2\times \frac{\mathrm{Precision}\times \mathrm{Recall}}{\mathrm{Precision}+\mathrm{Recall}}$$			

**Table 5:** Double Class Performance.

			Predicted		Accuracy (%)	Sensitivity (%)	Specificity (%)	f- Score (%)
			A	B				
**A-B**	Original	A	97	3	95.50	94.17	96.90	95.50
		B	6	94				
			**Predicted**					
			A	C	96.50	98.94	94.28	96.41
**A-C**	Original	A	94	6				
		C	1	99				
			**Predicted**					
			A	D	100	100	100	100
**A-D**	Original	A	100	0				
		D	0	100				
			**Predicted**					
			A	E	99.50	99.00	100	99.50
**A-E**	Original	A	100	0				
		E	1	99				
			**Predicted**					
			B	C	99.00	99.00	99.00	99.00
**B-C**	Original	B	99	1				
		C	1	99				
			**Predicted**					
			B	D	100	100	100	100
**B-D**	Original	B	100	0				
		D	0	100				
			**Predicted**					
			B	E	99.50	100	100	99.50
**B-E**	Original	B	100	0				
		E	1	99				
			**Predicted**					
			C	D	80.00	75.86	85.71	81.48
**C-D**	Original	C	88	12				
		D	28	72				
			**Predicted**					
			C	E	98.50	98.01	98.98	98.50
**C-E**	Original	C	99	1				
		E	2	98				
			**Predicted**					
			D	E	98.50	98.01	98.98	98.50
**D-E**	Original	D	99	1				
		E	2	98				

**Table 6:** Triple Class Performance.

			**Predicted**			**Accuracy (%)**	**Sensitivity (%)**	**Specificity (%)**	**f- Score (%)**
			**A**	**B**	**C**				
**A-B-C**	**Original**	A	92	3	5	95.00	92.00	96.50	92.46
		B	5	95	0	97.26	95.00	94.44	95.95
		C	2	0	98	97.60	98.00	97.39	96.55
			**Predicted**			**Accuracy (%)**	**Sensitivity (%)**	**Specificity (%)**	**f- Score (%)**
			**A**	**B**	**D**				
**A-B-D**	**Original**	A	96	2	2	96.99	96.00	97.48	95.52
		B	4	95	1	97.64	95.00	98.98	96.44
		D	1	0	99	98.63	99.00	98.45	98.01
			**Predicted**			**Accuracy (%)**	**Sensitivity (%)**	**Specificity (%)**	**f- Score (%)**
			**A**	**B**	**E**				
**A-B-E**	**Original**	A	96	4	0	96.30	96.96	95.97	94.58
		B	5	95	0	96.63	95.00	97.46	95.00
		E	3	1	96	98.62	96.00	100	97.95
			**Predicted**			**Accuracy (%)**	**Sensitivity (%)**	**Specificity (%)**	**f- Score (%)**
			**A**	**C**	**D**				
**A-C-D**	**Original**	A	94	4	2	95.65	94.00	96.59	94.00
		C	2	87	11	88.88	87.00	89.84	84.05
		D	1	16	83	89.79	83.00	93.29	94.69
			**Predicted**			**Accuracy (%)**	**Sensitivity (%)**	**Specificity (%)**	**f- Score (%)**
			**A**	**C**	**E**				
**A-C-E**	**Original**	A	96	4	0	97.18	96.00	97.82	96.00
		C	3	97	0	93.55	97.00	91.79	91.07
		E	1	16	83	94.19	83.00	100	90.71
			**Predicted**			**Accuracy (%)**	**Sensitivity (%)**	**Specificity (%)**	**f- Score (%)**
			**B**	**C**	**D**				
**B-C-D**	**Original**	B	98	1	1	98.56	98.00	98.87	98.00
		C	1	91	8	91.94	91.00	92.42	88.34
		D	1	14	85	91.94	85.00	95.45	87.62
			**Predicted**			**Accuracy (%)**	**Sensitivity (%)**	**Specificity (%)**	**f- Score (%)**
			**B**	**C**	**E**				
**B-C-E**	**Original**	B	100	0	0	98.99	100	98.49	98.52
		C	1	99	0	99.32	99.00	99.49	99.00
		E	2	1	97	98.99	97.00	100	98.47
			**Predicted**			**Accuracy (%)**	**Sensitivity (%)**	**Specificity (%)**	**f- Score (%)**
			**B**	**D**	**E**				
**B-D-E**	**Original**	B	100	0	0	98.98	100	98.47	98.52
		D	1	98	1	98.65	98.00	98.98	98.00
		E	2	2	96	98.32	96.00	99.49	97.46
			**Predicted**			**Accuracy (%)**	**Sensitivity (%)**	**Specificity (%)**	**f- Score (%)**
			**D**	**C**	**E**				
**D-C-E**	**Original**	D	82	17	1	89.71	82.00	93.95	84.97
		C	10	88	2	84.89	88.00	83.83	79.63
		E	1	16	83	92.67	83.00	98.26	89.24
			**Predicted**			**Accuracy (%)**	**Sensitivity (%)**	**Specificity (%)**	**f- Score (%)**
			**A**	**D**	**E**				
**A-D-E**	**Original**	A	100	0	0	99.00	100	98.50	98.52
		D	2	98	0	99.33	98.00	100	98.98
		E	1	0	99	99.66	99.00	100	99.49

**Table 7:** Quaternary Class Performance.

			**Predicted**				**Accuracy (%)**	**Sensitivity (%)**	**Specificity (%)**	**f- Score (%)**
			**A**	**C**	**D**	**E**				
**A-C-D-E**	**Original**	**A**	98	1	1	0	96.79	98.00	96.35	94.23
		**C**	4	81	15	0	92.58	81.00	95.56	84.81
		**D**	5	9	85	1	91.87	85.00	94.21	84.15
		**E**	1	0	1	98	99.00	98.00	99.62	98.49
			**Predicted**				**Accuracy (%)**	**Sensitivity (%)**	**Specificity (%)**	**f- Score (%)**
			**B**	**C**	**D**	**E**				
**B-C-D-E**	**Original**	**B**	98	1	1	0	98.65	98.00	98.89	97.51
		**C**	1	86	13	0	92.89	86.00	95.23	86.00
		**D**	0	13	84	3	92.42	84.00	95.27	84.84
		**E**	2	0	0	98	98.65	98.00	98.89	97.51

**Table 8:** Five Class Performance.

			Predicted					Accuracy (%)	Sensitivity (%)	Specificity (%)	f-Score (%)
			A	B	C	D	E				
**A-B-C-D-E**	**Original**	**A**	95	3	0	0	2	97.90	95.00	98.67	95.00
		**B**	3	97	0	0	0	98.52	97.00	98.93	96.51
		**C**	1	1	87	11	0	95.31	87.00	97.44	88.32
		**D**	0	0	10	90	0	95.70	90.00	97.17	89.55
		**E**	1	0	0	0	99	99.36	99.00	99.46	98.50

**Table 9:** The Reported Works on the Classification of Bonn EEG Signal.

Study	Method Used	Datasets	Success (%)
[14]	TQWT-Based Multi-Scale k-NN entropy	A-E	100
[19]	DWT + SE/SD/BP + KNN/SVM	A-E	100
[39]	Wavelet Transform + PCA, GBM, RF, and SVM	A-E	100
[40]	LMD + GA + SVM	A-E	100
[21]	L1-Penalized Robust Regression + RF	A-E	100
[13]	DWT + Fuzzy Approximate Entropy + SVML	A-E	100
[41]	FFT and Decision Tree	A-E	98.70
[42]	Wavelet Transform, Phase, Euclid Distance	A-E	98.17
[43]	Artificial Neural Networks	A-E	97.50
[11]	1-D-LBP and Bayes Net	A-D	99.50
[44]	LMD + GA-SVM	D-E	98.10
[39]	Wavelet Transform + PCA, GBM, RF, and SVM	D-E	98.10
[14]	TQWT- K-NN Entropy	D-E	98.00
[15]	CEEMDAN + RF	D-E	98.00
[40]	DTCWT + GRNN	D-E	98.00
[45]	Weighted Permutation Entropy + SVM	D-E	96.50
[13]	DWT + Fuzzy Approximate Entropy + SVML	D-E	95.85
[44]	LMD+GA+SVM	A-D-E	98.47
[13]	DWT + Fuzzy Approximate Entropy + SVML	A-D-E	95.67
[11]	1D-LBPand Bayes Net (LBP all)	A-D-E	95.67
[16]	MEMD +ANN	A-B-C-D-E	87.2%
In this Study	CNN + Scalogram	A-E	99.50
	CNN + Scalogram	A-D	100
	CNN + Scalogram	D-E	98.50
	CNN + Scalogram	A-D-E	99.00
	CNN + Scalogram	A-B-C-D-E	93.60

## Abbreviations

The following abbreviations are used in this manuscript:

CNN	Convolutional Neural Network
CWT	Continuous Wavelet Transform
TQWT	Tunable-Q Wavelet Transform
DWT	Discrete Wavelet Transform
SE	Shannon Entropy
SD	Standard Deviation
BP	Band Power
SVM	Support Vector Machine
LMD	Local Mean Decomposition
GA	Genetic Algorithm
RF	Random Forest
SVML	Linear Basis Function Based Support Vector Machine
FFT	Fast Fourier Transform
1-D-LBP	One Dimensional Local Binary Pattern
CEEMDAN	Complete Ensemble Empirical Mode Decomposition with Adaptive Noise
DTCWT	Dual-Tree Complex Wavelet Transform
GRNN	General Regression Neural Network
MEMD	Multivariate Empirical Mode Decomposition
EMD	Empirical Mode Decomposition
PCA	Principal Component Analysis
LS-SVM	Least Squares Version of Support Vector Machine
ME	Mixture of experts
IMFs	Intrinsic Mode Functions
ApEn	Approximate entropy
SDAE	Stacked Denoising Autoencoders
